# Structural properties and anti-dermatitis effects of flavonoids-loaded gold nanoparticles prepared by *Eupatorium japonicum*

**DOI:** 10.3389/fphar.2022.1055378

**Published:** 2022-10-31

**Authors:** Xing Yue Xu, Sung-Kwon Moon, Jin-Kyu Kim, Woo Jung Kim, Yeon-Ju Kim, Hoon Kim

**Affiliations:** ^1^ Graduate School of Biotechnology, And College of Life Science, Kyung Hee University, Yongin, South Korea; ^2^ Beijing Key Laboratory of Traditional Chinese Medicine Basic Research on Prevention and Treatment for Major Diseases, Experimental Research Center, China Academy of Chinese Medical Sciences, Beijing, China; ^3^ Department of Food and Nutrition, Chung Ang University, Anseong, South Korea; ^4^ Biocenter, Gyeonggido Business and Science Accelerator, Suwon, South Korea

**Keywords:** biosynthesized nanomaterial, plant-loaded nanoparticle, inflammatory skin disease, chemokine, HaCaT, secondary metabolites

## Abstract

Recently, green synthesis-based nanoformulations using plants or microorganisms have attracted great interest because of their several advantages. Nanotechnology-based biological macromolecules are emerging materials with potential applications in cosmetics and medications for ameliorating and treating inflammatory skin diseases (ISDs). *Eupatorium japonicum* (EJ), a native Korean medicinal plant belonging to the family Asteraceae, has been traditionally used to prepare prescriptions for the treatment of various inflammatory diseases. EJ-based gold nanoparticles (EJ-AuNPs) were biosynthesized under optimal conditions and characterized their physicochemical properties using various microscopic and spectrometric techniques. Additionally, the effects of EJ-AuNPs on ISDs as well as their underlying mechanisms were investigated in the tumor necrosis factor-α/interferon-γ (T+I)-induced skin HaCaT keratinocytes. The MTT and live/dead cell staining assays showed that EJ-AuNP treatment was considerably safer than EJ treatment alone in HaCaT cells. Moreover, EJ-AuNP treatment effectively suppressed the production of T+I-stimulated inflammatory cytokines (RANTES, TARC, CTACK, IL-6, and IL-8) and intracellular reactive oxygen species, and such EJ-driven anti-inflammatory effects were shown to be associated with the downregulation of intracellular mitogen-activated protein kinase and nuclear factor-κB signaling pathways. The present study provides preliminary results and a valuable strategy for developing novel anti-skin dermatitis drug candidates using plant extract-based gold nanoparticles.

## Introduction

Inflammation is a pivotal biological reaction caused by irritation against harmful stimuli, and is a protective response involving immune cells, blood vessels, and inflammatory mediators (Chen et al., 2017). However, chronic inflammation due to persistent uncontrolled acute inflammation may contribute to multiple chronic diseases, such as allergies, cardiovascular and bowel diseases, diabetes, metabolic syndrome, arthritis, cancer, and autoimmune diseases (Ghasemian et al., 2016; Chen et al., 2017). Skin inflammation, also called dermatitis, is an inflammatory reaction that occurs in the skin tissue and is accompanied by symptoms, such as itchiness, redness, heat, and rash. Persistent pathological inflammatory skin diseases (ISDs) include atopic dermatitis, allergic dermatitis, contact dermatitis, seborrheic dermatitis, stasis dermatitis, etc. (Albanesi and Pastore, 2010; Carretero et al., 2016). Due to the rapid climate change and environmental pollution, an increasing number of people are suffering from chronic dermatitis, which is emerging as a serious social problem (Drakaki et al., 2014).

Recently, nanotechnology-based therapeutics have been considered as emerging materials with potential applications in cosmetics and medications to ameliorate and treat ISDs (Wiesenthal et al., 2011; Puglia and Bonina, 2012; Kakkar et al., 2019). Since the first report on the topical application of nanoparticles in the 1980s, various nanoformulations have been applied to treat ISDs (Kakkar et al., 2019; Campos et al., 2021). Despite the widespread study of nanoformulations, major concerns regarding their cytotoxicity have limited their industrial application (Ahmed et al., 2016; Bahadar et al., 2016). To overcome these limitations, green synthesis-based nanoparticles using plants or microorganisms are of great interest owing to their several advantages over other nanoformulations, such as relatively high biocompatibility and reproducibility, easy and simple preparation procedures, low cost, and eco-friendly nature (Krishnaswamy et al., 2014; Ahmed and Ikram, 2015). Especially, gold nanoparticles (AuNPs) have gained the most attention in various fields because of their several advantages, including synthesis compatibility, biocompatibility, low toxicity, and detection capability (Dykman and Khlebtsov, 2012; Wang et al., 2021). However, as only a few studies have reported their efficacy on the skin, more fundamental research on plant-based gold nanoparticles is required for dermal application as they are known to have various benefits over traditional topical treatments, such as improved skin permeation, regulated drug delivery, and enhanced therapeutic effects (Abdel-Mottaleb et al., 2014).

A native Korean medicinal plant, *Eupatorium japonicum* (EJ), which belongs to the Asteraceae family, is found primarily in Northeast Asia, including Korea, China, and Japan. EJ is traditionally used to prepare prescriptions to treat various digestive diseases, nausea, vomiting, dyspepsia, and diarrhea (Shin et al., 2018). Recently, the therapeutic efficacy of EJ against inflammatory reactions and its underlying mechanisms of action were investigated in RAW 264.7 monocytic, 293T kidney, and rheumatoid arthritis fibroblast-like synovial cells (Gu et al., 2014; Dai et al., 2020; Phan et al., 2021), and it was found that EJ may be useful for controlling and treating ISDs. However, the effects of EJ-mediated nanoparticles on ISDs have not yet been examined.

Therefore, the present study aimed to prepare novel AuNPs using EJ extracts and identify their physicochemical characteristics. In addition, we aimed to explore the efficacy of EJ-based AuNPs in ISDs and their underlying molecular mechanism using an inflammation-induced human keratinocyte model.

## Materials and methods

### Harvest and extraction of *Eupatorium japonicum* leaf tissue

The leaf tissue of wild EJ plant was harvested from northern Gyeonggi, adjacent to the demilitarized zone in Korea. The plant was identified by Dr. J. K. Kim, a senior researcher at Gyeonggido Business and Science Accelerator, Gyeonggi Biocenter (Suwon, Korea). A voucher specimen was deposited in the same department as described above. Dried leaves were immersed in five volumes (w/v) of 70% ethanol and extracted at 20°C–25°C for 3 days. A polyester filtering cloth (20 μm; Hyundai Micro, Anseong, Korea) was used to filter the extract, which was then concentrated using a rotary evaporator (Buchi Korea Inc., Gwangmyeong, Korea). After drying using a freeze-drier (Ilshin Biobase, Daejeon, Korea), the 70% ethanol extract of EJ leaves was prepared with an extraction yield of 21.1%.

### Qualitative determination of major *phytochemicals* by ultra-performance liquid chromatography–tandem mass spectrometry analysis

A UPLC-MS system (LTQ Orbitrap XL, Thermo Electron, Waltham, MA, United States) was used for the qualitative determination of the major phytochemicals in EJ, according to a previously described analytical method (Kim et al., 2021). The detailed analytical conditions are listed in Supplementary Table S1.

### Biosynthesis of optimized EJ-AuNPs

To establish the optimal conditions for AuNP biosynthesis from EJ, four parameters were monitored: EJ concentration, HAuCl_4_•3H_2_O concentration, reaction temperature, and time. Briefly, distilled water (1 ml) containing HAuCl_4_•3H_2_O (0.5–2.5 mM, 0.5 mM interval) was added to dried EJ (1–6 mg, 1 mg interval), and the mixture was incubated at the designated temperature (30°C–70°C, 10°C interval) for 20–50 min (10 min interval). After the reaction, the color change and absorbance were measured with the eye and using a UV-Vis spectrometer (Agilent Technologies, Inc., Santa Clara, CA, United States), respectively, to ascertain the optimal conditions for the synthesized nanoparticles. To remove the soluble materials, the mixture was centrifuged (12,000 rpm, 20 min), and the precipitated particles were washed with distilled water. This process was repeated four more times to purify the particles. The particles were finally dried using a freeze-drier (Ilshin Biobase, Daejeon, Korea) to obtain AuNPs biosynthesized from EJ (EJ-AuNPs).

### Physicochemical analysis of *Eupatorium japonicum*-AuNPs

The thermal stability of the EJ-AuNPs was measured by thermogravimetric analysis (TGA) (TGA/DSC 1; Yeonjin S-Tech Co., Seoul, Korea) at 30°C–600°C. To identify the functional groups on the surface of the EJ-AuNPs, Fourier transform infrared (FT-IR) spectroscopy (PerkinElmer Inc., Waltham, MA, United States) was used in the range 500–4000 cm^−1^. The particle sizes and size distribution of the EJ-AuNPs were measured using a dynamic light scattering (DLS) particle size analyzer (Otsuka Electronics, Shiga, Japan) at a size range of 1–1000 nm. Field-emission-transmission electron microscopy (FE-TEM; JEM-2100F, JEOL, Ltd., Tokyo, Japan) coupled with selected area electron diffraction (SAED) and energy-dispersive X-ray spectrometry (EDX) was used at a voltage of 200 kV to measure the particle size, microscopic morphology, elemental composition, and crystalline nature of the EJ-AuNPs. A powered X-ray diffractometer (XRD; D8 Advance, Bruker, Karlsruhe, Germany) was used with 1.54 Å CuKα radiation to confirm the purity and crystalline nature of the EJ-AuNPs.

### HaCaT cell culture and evaluation of cytotoxic effect

Human epidermal keratinocytes (HaCaT; CLS GmbH, Eppelheim, Germany) were cultured in Dulbecco’s modified Eagle’s medium (DMEM; GenDEPOT, Katy, TX, United States) containing 10% fetal bovine serum (FBS; GenDEPOT), 100 U penicillin, and 100 μg/ml streptomycin (GenDEPOT). The cells were then incubated in a humidified incubator with 5% carbon dioxide (CO_2_)/95% air. Cells (1 × 10^4^ cells) were plated in a 96-well plate (SPL Life Sciences, Pocheon, Korea) and stabilized for 24 h. The dried EJ-AuNPs or EJ was diluted with serum-free medium (SFM) at concentrations of 25, 50, and 100 μg/ml. After the cells were washed twice with phosphate-buffered saline (PBS), diluted EJ-AuNPs solution was added to the cells and incubated for a further 24 h. To compare the cytotoxic effect of EJ-AuNPs, only SFM and commercial dexamethasone (20 μg/ml)-containing SFM were added to the cells. The cytotoxic effects of EJ-AuNPs against HaCaT cells were measured using a conventional 3-(4,5-cimethylthiazol-2-yl)-2,5-diphenyl tetrazolium bromide (MTT) solution (Sigma-Aldrich, St. Louis, MO, United States) and a live/dead cell staining kit (Thermo Fisher Scientific, Cambridge, MA, United States), according to the manufacturer’s instructions.

### Reactive oxygen species staining

HaCaT cells (2 × 10^5^ cells) were seeded in a 6-well plate (SPL Life Sciences) and stabilized for 24 h. After the cells were washed twice with PBS, they were treated with fresh SFM containing EJ-AuNPs for 1 h. To compare the levels of ROS in EJ-AuNP-treated cells, only SFM and commercial dexamethasone (20 μg/ml)-containing SFM were added to the cells. Subsequently, the cells were treated with a recombinant protein mixture comprising 10 ng/ml tumor necrosis factor-alpha (TNF-α; 210-TA-100/CF, R&D Systems, Minneapolis, MN, United States) and 10 ng/ml interferon-gamma (IFN-γ; 285-IF-100/CF, R&D Systems) (T+I) for 24 h. After rinsing twice with PBS, the cells were stained using a cellular ROS assay kit (ab113851; Abcam, Cambridge, United Kingdom) according to the manufacturer’s instructions. Intracellular ROS levels were visualized using a Leica DM IRB fluorescence microscope (Leica Microsystems, Wetzlar, Germany) and quantified using the GraphPad software (Prism 8; San Diego, CA, United States)

### Quantitative reverse transcription-polymerase chain reaction

HaCaT cells (5 × 10^5^ cells) were seeded onto a 60 mm dish (SPL Life Sciences) and stabilized for 24 h. After the cells were washed twice with PBS, fresh SFM containing EJ-AuNPs was added to the cells for 1 h. To compare the gene expression levels in EJ-AuNP-treated cells, SFM and commercial dexamethasone (20 μg/ml)-containing SFM were also added to the cells. Subsequently, the cells were treated with T+I mixture for 24 h. After rinsing twice with PBS, total RNA was extracted using the TRIzol reagent (Invitrogen, Carlsbad, CA, United States) and quantified on a nanodrop plate using a microplate spectrophotometer (Epoch, BioTek Instruments, Winooski, VT, United States). Equal amounts of total RNA were reverse-transcribed using the AmfiRivert cDNA synthesis kit (GenDEPOT), and qRT-PCR was performed using the AmfiSure qGreen Q-PCR master mix (GenDEPOT) and Rotor-gene Q real-time PCR detection system (Qiagen, Hilden, Germany) with SYBR Premix Ex Taq^TM^ II (TaKaRa Bio Inc., Kusatsu, Japan). All primers were designed and provided by Macrogen (Seoul, Korea). The gene-specific primer sequences used in this study are listed in Supplementary Table S2. The level of target gene expression was calculated and normalized against the expression level of the endogenous control gene, glyceraldehyde-3-phosphate dehydrogenase (*GAPDH*), using formula 2^−ΔΔ^Ct.

### Enzyme-linked immunosorbent assay

HaCaT cells (1 × 10^4^ cells) were seeded in a 96-well plate (SPL Life Sciences) and stabilized for 24 h. After the cells were washed twice with PBS, they were treated with fresh SFM containing EJ-AuNPs for 1 h. To compare the cytokine secretion to EJ-AuNP-treated cells, only SFM and commercial dexamethasone (20 μg/ml)-containing SFM were added to the cells. Subsequently, the cells were treated with the T+I mixture for 24 h. The cell-free culture medium was collected, and pro-inflammatory cytokines, including interleukin (IL)-6 (555220; BD Biosciences, Newark, DE, United States), IL-8 (555,244; BD Biosciences), and thymus and activation-regulated chemokine (TARC) (DY364; R&D Systems), were measured using ELISA, according to the manufacturer’s instructions.

### Immunoblotting analysis

The HaCaT cell culture method and sample treatment procedures were carried out according to the procedures described in Section 2.6. After rinsing twice with PBS, total protein was isolated from the cells using the Pierce RIPA buffer reagent (Thermo Fisher Scientific) containing protease inhibitors (GenDEPOT) and quantified using a bicinchoninic acid protein assay kit (Thermo Fisher Scientific). Equal amounts of total protein were separated on a 10% sodium dodecyl sulfate-polyacrylamide gel, and the separated proteins were transferred from the gel to a polyvinylidene fluoride membrane (Thermo Fisher Scientific). The membrane was blocked with PBST containing 5% skim milk at 20°C–25°C for 2 h and washed thrice with PBST. The membranes were incubated with primary antibodies against p38 mitogen-activated protein kinase (p38; #8690), c-Jun N-terminal kinase (JNK; #9252), extracellular signal-regulated kinase (ERK1/2; #4695), nuclear factor kappa-light-chain-enhancer of activated B cells p65 (p65; 8242), nuclear factor kappa-light-chain-enhancer of activated B cells inhibitor alpha (IκBα; #4814), β-actin (#3700), p-p38 (#4511), p-JNK (#4668), p-ERK (#4370), p-p65 (#3033), and p-IκBα (#9246) at 4°C overnight. After washing three times with PBST, the membrane was incubated with horseradish peroxidase-conjugated secondary antibody against anti-mouse/rabbit IgG (#98164) at 20°C–25°C for 1 h. All antibodies were obtained from Cell Signaling Technology (Danvers, MA, United States). The membrane was rinsed five times with PBST, and the protein blots were visualized using the West-Q Pico ECL Solution (GenDEPOT). The expression level of each target protein was quantified using the ImageJ software available in online website (https://imagej.nih.gov/ij/).

### Statistical analysis

All experiments were performed in triplicate, and the results are expressed as the mean ± standard deviation. Statistical analyses were performed using PASW Statistics 18 (IBM Co., Armonk, NY, United States). Statistical comparisons between two groups were conducted using the Student’s *t*-test, and *p* < 0.05, *p* < 0.01, and *p* < 0.001 were considered statistically significant at different levels.

## Results

### Putative identification of major phytochemicals in the EJ extract

**FIGURE 1:**
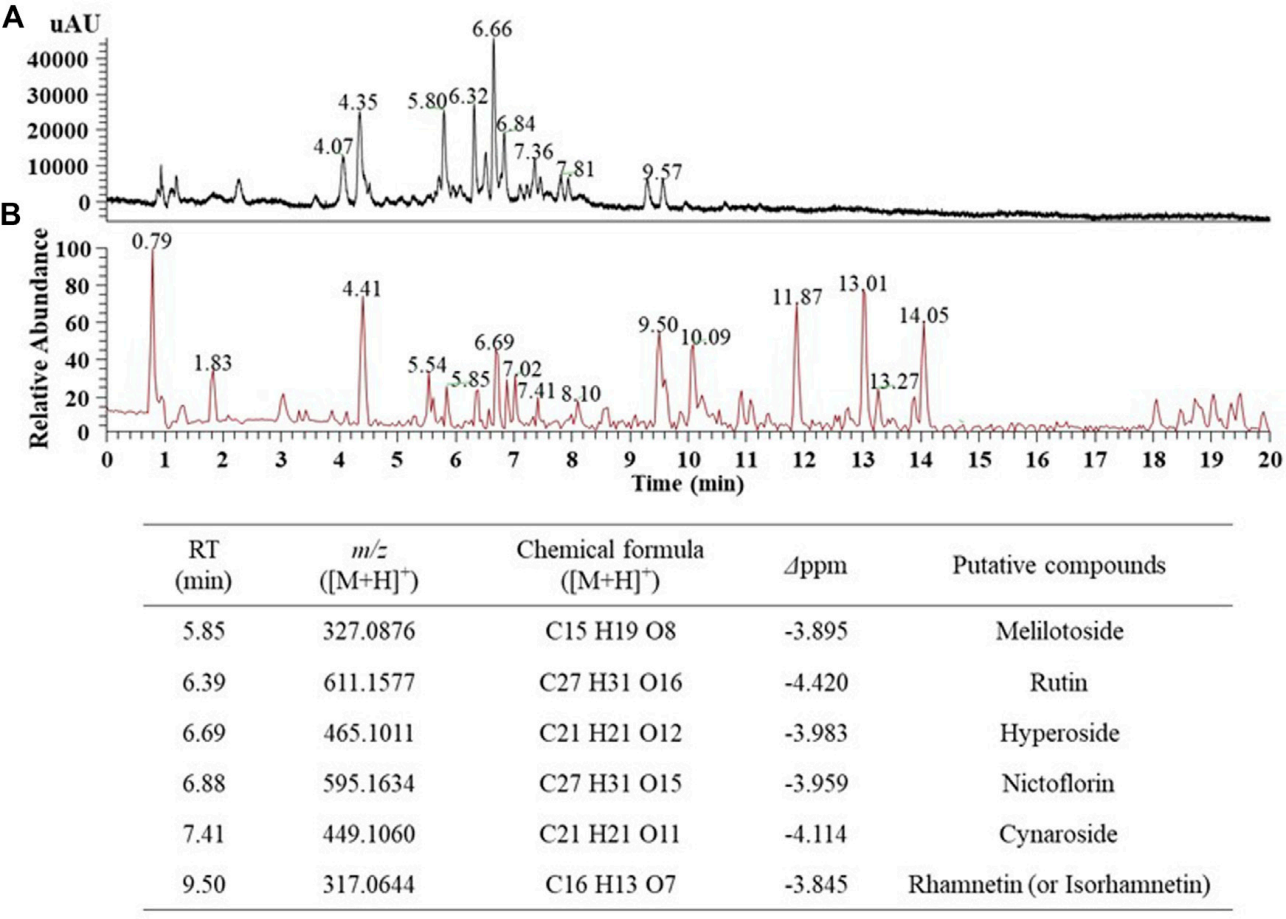
UPLC-MS chromatogram of the 70% ethanol extract of *E. japonicum* (EJ). **(A)** Photo diode array (PDA) chromatogram, **(B)** Total ion chromatogram (TIC), and putative identification of six major phytochemicals in EJ as listed in Table. EJ (10 mg/ml) was introduced into the UPLC system, and separation and detection conditions were described in Supplementary Table S1.

**FIGURE 2:**
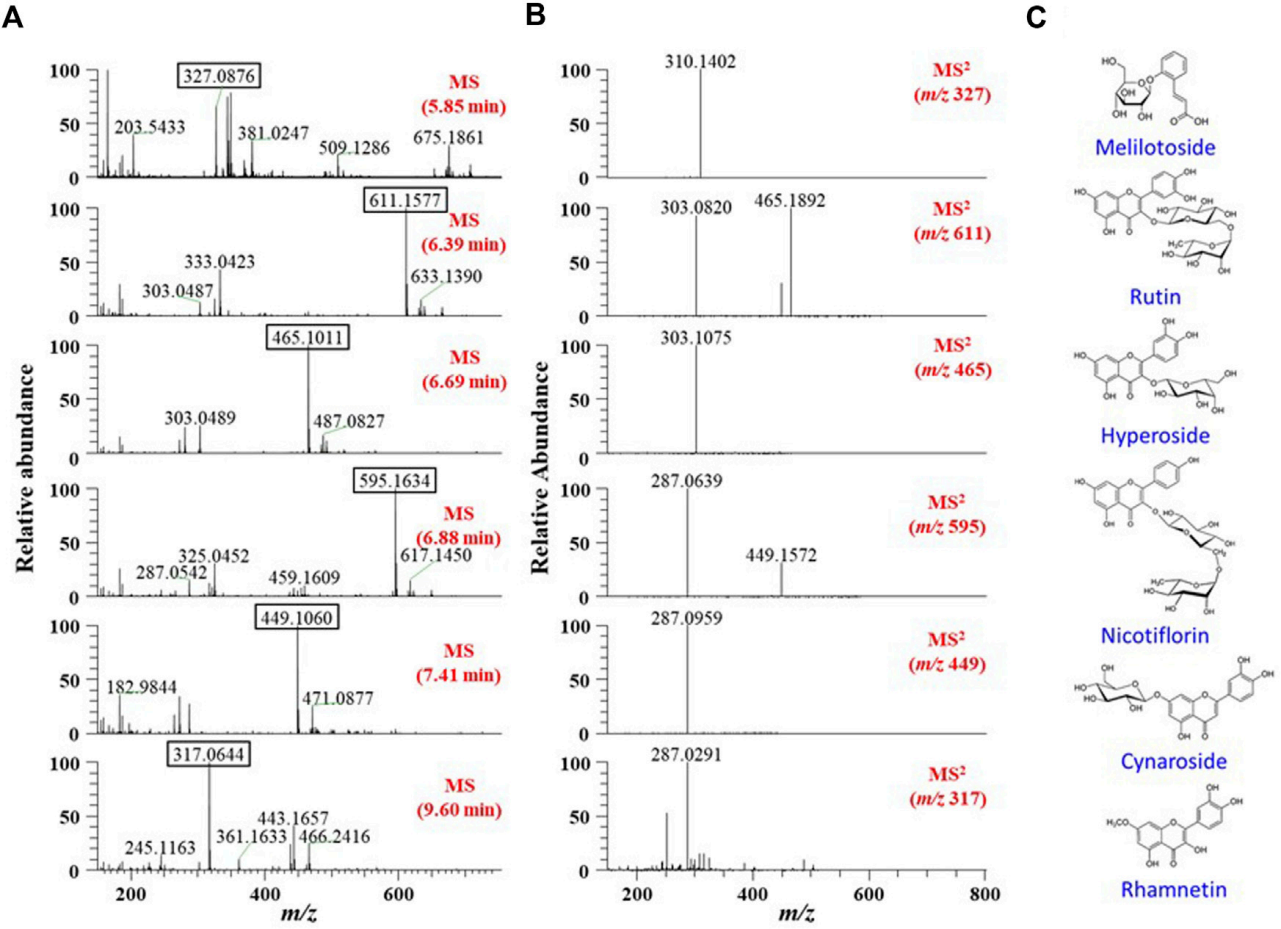
**(A)** MS and **(B)** MS/MS spectra of six major peaks identified in TIC (Figure 1). Each peak in the spectra was identified using a natural-product database available online and an in-house MS/MS spectral library. **(C)** Chemical structures of the identified six phytochemicals in EJ.

First, we aimed to determine the major phytochemicals present in the EJ extract using UPLC-MS/MS analysis. PDA and BPC of the EJ extract are shown in Figures 1A,B, respectively. The molecular weight and ionization pattern of each peak on the BPC were further analyzed by MS (Figure 2A) and MS/MS (Figure 2B) to determine the identity of the putative compounds by retrieving an in-house spectral library and a web-based database. The results showed that an ion peak at 5.85 min in BPC was confirmed as *m/z* 327.0876 in the positive ionization mode of MS, and MS/MS provided information on the putative identification of melilotoside (CAS No. 618–67-7) by determining its product ion at *m/z* 310.1402 ([M+H]^+^). Using the same process, five peaks (6.39, 6.69, 6.88, 7.41, and 9.50 min) on the BPC were putatively identified as rutin (quercetin-3-O-rutinoside), hyperoside (quercetin 3-*O*-β-galactoside), nictoflorin (kaempferol-3-*O*-β-rutinoside), cynaroside (luteolin 7-*O*-β-D-glucoside), and rhamnetin (quercetin 7-methyl ether) by their values of *m/z* 611.1580 ([M+H]^+^), 465.1009 ([M+H]^+^), 595.1637 ([M+H]^+^), 449.1060 ([M+H]^+^), and 317.0644 ([M+H]^+^), respectively. The chemical structures of aforementioned compounds were illustrated in Figure 2C.

### Establishment of optimized conditions for EJ-AuNP synthesis

**FIGURE 3:**
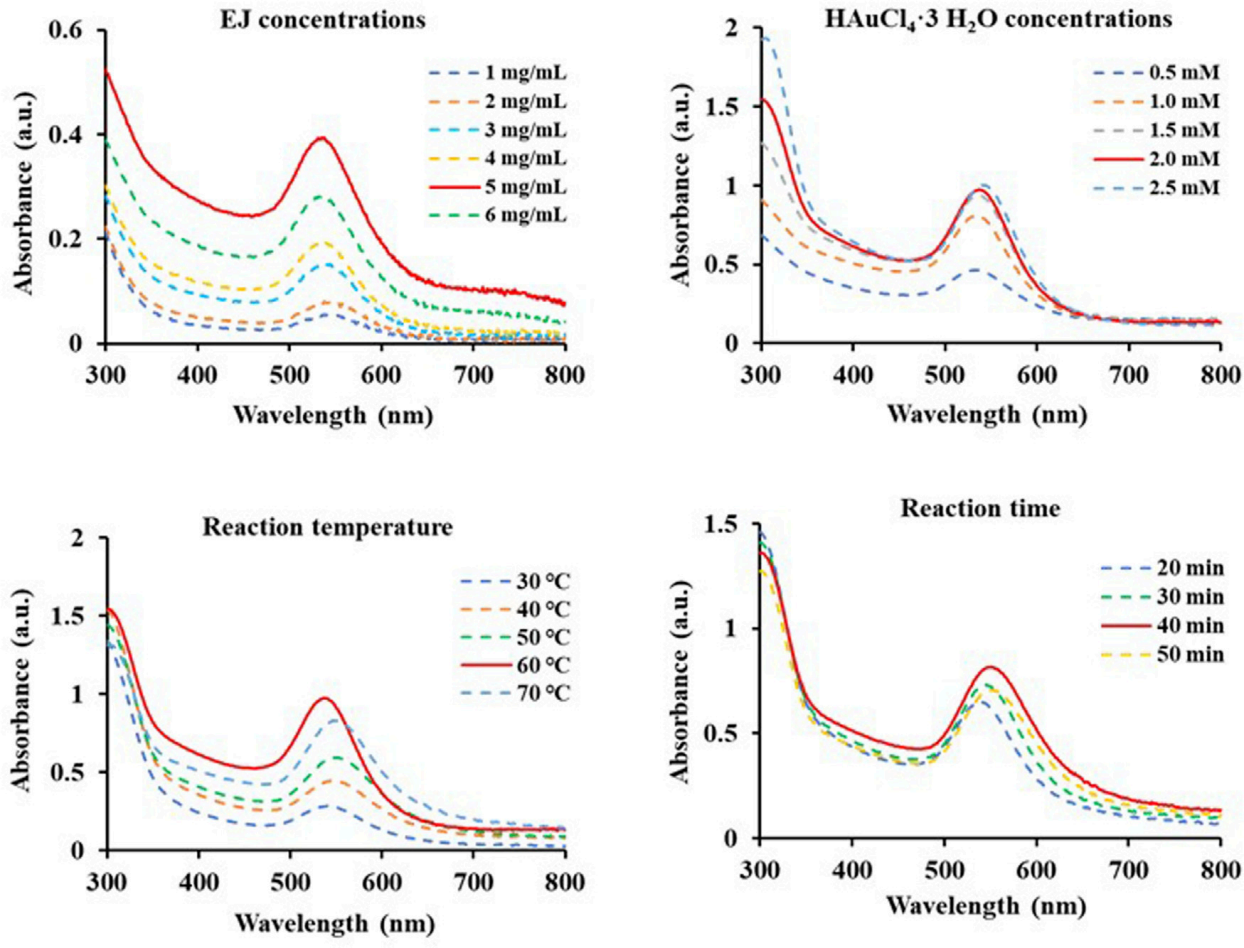
Scanning spectra at absorbances of 300–800 nm of diverse EJ-based gold nanoparticles (EJ-AuNPs) synthesized under various conditions depending on EJ concentrations (1–6 mg/ml), HAuCl_4_•3H_2_O concentrations (0.5–2.5 mM), reaction temperatures (30°C–70°C), and time (20–50 min). The red solid line in each Figure indicates the optimal condition.

Various conditions to establish optimal conditions for the biosynthesis of EJ-AuNPs were monitored in terms of EJ concentrations (1–6 mg/ml), gold salt concentrations (0.5–2.5 mM), reaction temperatures (30°C–70 °C), and reaction times (20–50 min) using the UV-Vis spectrometric method. Optimal conditions for EJ-AuNP biosynthesis were established by confirming the absorption spectra with the highest peak (solid red line): 5 mg/mL EJ concentration (Figure 3A), 2 mM gold salt (Figure 3B), and reaction at 60°C for 40 min (Figures 3C,D). EJ-AuNPs were synthesized under optimal conditions and used for subsequent experiments.

### Characterization of the physiochemical properties of EJ-AuNPs

**FIGURE 4:**
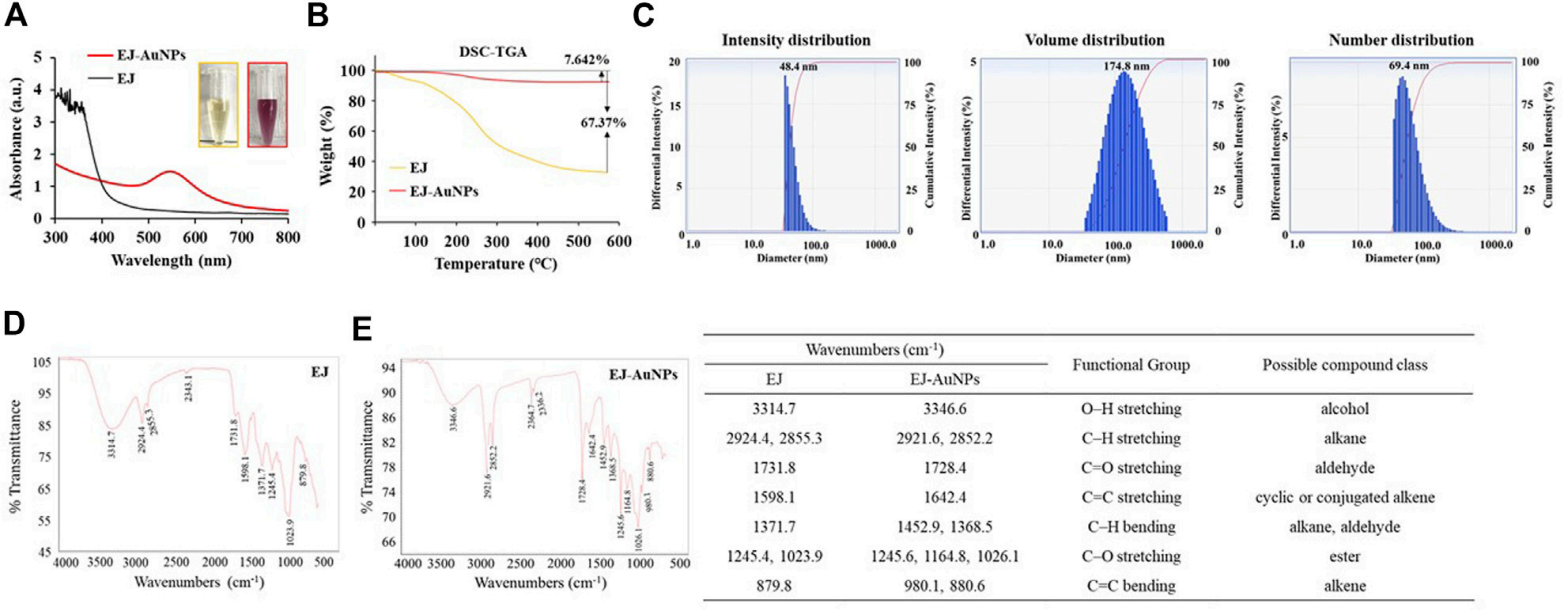
Determination of the physicochemical properties of EJ-AuNPs. Comparison of **(A)** UV-Vis spectrum and **(B)** thermogravimetric thermogram of EJ and EJ-AuNPs. **(C and D)** Fourier-transform infrared (FT-IR) spectra of EJ and EJ-AuNPs. **(E)** Dynamic light scattering (DLS) spectra of EJ-AuNPs.

Figure 4A shows the UV-Vis spectra of EJ-AuNPs and EJ. Although no specific absorbance was observed for EJ, EJ-AuNPs exhibited a robust peak at an absorbance of 547 nm. Given the visible color difference between EJ (transparent yellow) and EJ-AuNPs (dark purple), the absorbance spectral change seemed to be derived from the change in the surface plasmon band of the synthesized EJ-AuNPs. Figure 4B shows the TGA results which yield the oxidation temperature and residual mass of the sample, indicate the stability of the samples at a temperature range of 0°C–600°C. Following decomposition, the residual mass is considered as the ash contents for carbon-based materials (e.g., EJ), but it could be primarily inorganic nanomaterials, residual metal catalysts from synthesis, or impurities, within the sample, for cases of nanomaterials (Mansfield et al., 2014). During the thermal increase to 600°C, severe weight loss (–68.370%) was observed in EJ, but weak weight loss (–7.642%) occurred in EJ-AuNPs, suggesting that the synthesized EJ-AuNPs have better thermal stability than EJ. Furthermore, physicochemical stability of EJ-AuNPs after 1 month of biosynthesis were evaluated through visible observation, UV-VIS spectrum, and TEM analysis (Supplementary Figure S1), suggesting that EJ-AuNPs could be stable without any physicochemical changes at aforementioned condition (Tran et al., 2022). The size distribution profiles analyzed by DLS revealed that the mean intensity, volume, and number distributions of EJ-AuNPs were 48.4, 174.8, and 69.4 nm, respectively (Figure 4C). In order to identify functional groups which are responsible for the reduction of Au^3+^ cation and their capping and stabilization, FT-IR spectrum were compared between EJ and EJ-AuNPs (Figures 4D,E, respectively), and possible compound classes were tabulated. The results indicated that EJ and EJ-AuNPs have similar IR spectra each other. According to our in-house spectral library, the broad symmetrical stretching at 3314.7 cm^−1^ in EJ and 3346.6 cm^−1^ in EJ-AuNPs may indicate O–H stretching of the alcohol or N–H stretching of the amine groups. However, given the LC-MS results that flavonoid glycosides were the major components present in the EJ (Figure 1, Figure 2), it is believed that the aforementioned peaks are likely to be the groups of O–H stretching, rather than N–H stretching. The sharp and asymmetrical bands at 2924.4 cm^−1^ and 2855.3 cm^−1^ in EJ and 2921.6 cm^−1^ and 2852.2 cm^−1^ in EJ-AuNPs undoubtedly corresponded to the C–H stretching of alkane, and the peaks at 1731.8 cm^−1^ in EJ and 1728.4 cm^−1^ in EJ-AuNPs probably corresponded to the C=O stretching of the aldehyde (carbonyl) group. The absorption peak at 1642.4 cm^−1^ in EJ-AuNPs and 1598.1 cm^−1^ in EJ correspond to the stretching of alkene, and the peaks at 1371.7 cm^−1^ in EJ and 1452.9 cm^−1^ and 1368.5 cm^−1^ in EJ-AuNPs were associated with to the C–H bending of alkane or aldehyde. The several peaks at 1245–1023 cm^−1^ probably corresponded to the C–O stretching of ester, and the weak characteristic peaks at 879.8 cm-1 in EJ and 980.1 cm^−1^ and 880.6 cm^−1^ in EJ-AuNPs were maybe associated with the C=C bending of alkene.

**FIGURE 5:**
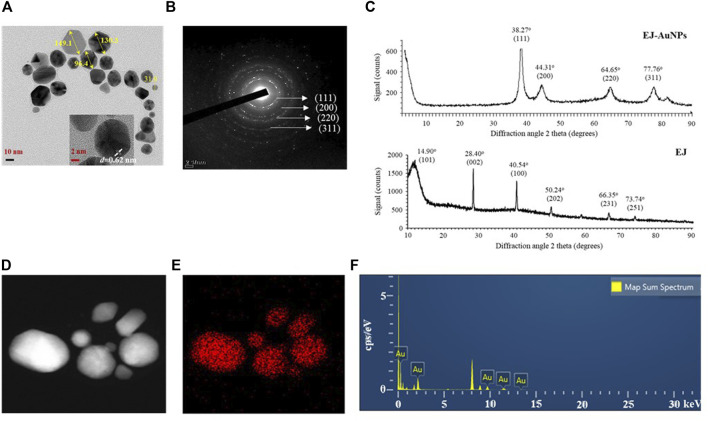
Physicochemical characteristics of EJ-AuNPs. **(A)** Transmission electron microscopy (TEM) for determining the surface morphology and particle sizes of EJ-AuNPs. **(B)** Selected area electron diffraction (SAED) pattern and **(C)** X-ray diffraction (XRD) spectrum for determining the crystalline structure of EJ-AuNPs and EJ. **(D)** Electron images of EJ-AuNPs obtained by energy dispersive X-ray (EDX) analysis. **(E)** Gold elemental distribution (red dots) of EJ-AuNPs obtained by EDX analysis. **(F)** EDX spectrum of EJ-AuNPs for identifying elemental distribution.

FE-TEM analysis was performed to identify the predominant shape, size, and crystalline structure of EJ-AuNPs. First, FE-TEM images showed that EJ-AuNPs had a particle size of 31.0–149.1 nm with surface morphologies predominantly of circular, spherical, and polygonal shapes (Figure 5A). To confirm the crystalline nature of the EJ-AuNPs, crystallographic techniques, such as SEAD and XRD, were introduced in this study. The SAED pattern revealed four ring features (111, 200, 220, and 311) observed in the lattice planes, confirming the crystalline structure of the EJ-AuNPs (Figure 5B). This is also verified by the XRD results of EJ-AuNPs, which show diffraction peaks at theta values of 38.27°, 44.31°, 64.65°, and 77.76°, which are calculated for the (111), (200), (220), and (311) planes, respectively, using Bragg’s equation (Figure 5C). Whereas, the XRD spectrum of EJ revealed well-resolved characteristic peaks at theta values of 14.90°, 28.40°, 40.54°, 50.24°, 66.35°, and 73.74°, corresponding to (101), (002), (100), (202), (231), and (251) planes, which are estimated to be amorphous structure with no obvious crystallization (Figure 5C). The purity of the EJ-AuNPs was determined by elemental mapping and EDX spectroscopy. As shown in Figures 5D,E, the distribution of gold elements (red dots) was clearly discernible within the nanoparticles, indicating that EJ-AuNPs were successfully synthesized with high purity. This is also consistent with the result of EDX spectroscopy (Figure 5F), in which only gold element peaks were observed in the synthesized EJ-AuNPs, suggesting that EJ-AuNPs were successfully synthesized without impurities. Excepting the Au peaks, other peaks without symbol observed at 1.9, 8.0, and 8.9 keV were confirmed as copper grids which is used as sample support on the EDX spectrum (Dhandapani et al., 2021).

### Effect of EJ and EJ-AuNPs on cytotoxicity in T+I-induced HaCaT cells

**FIGURE 6:**
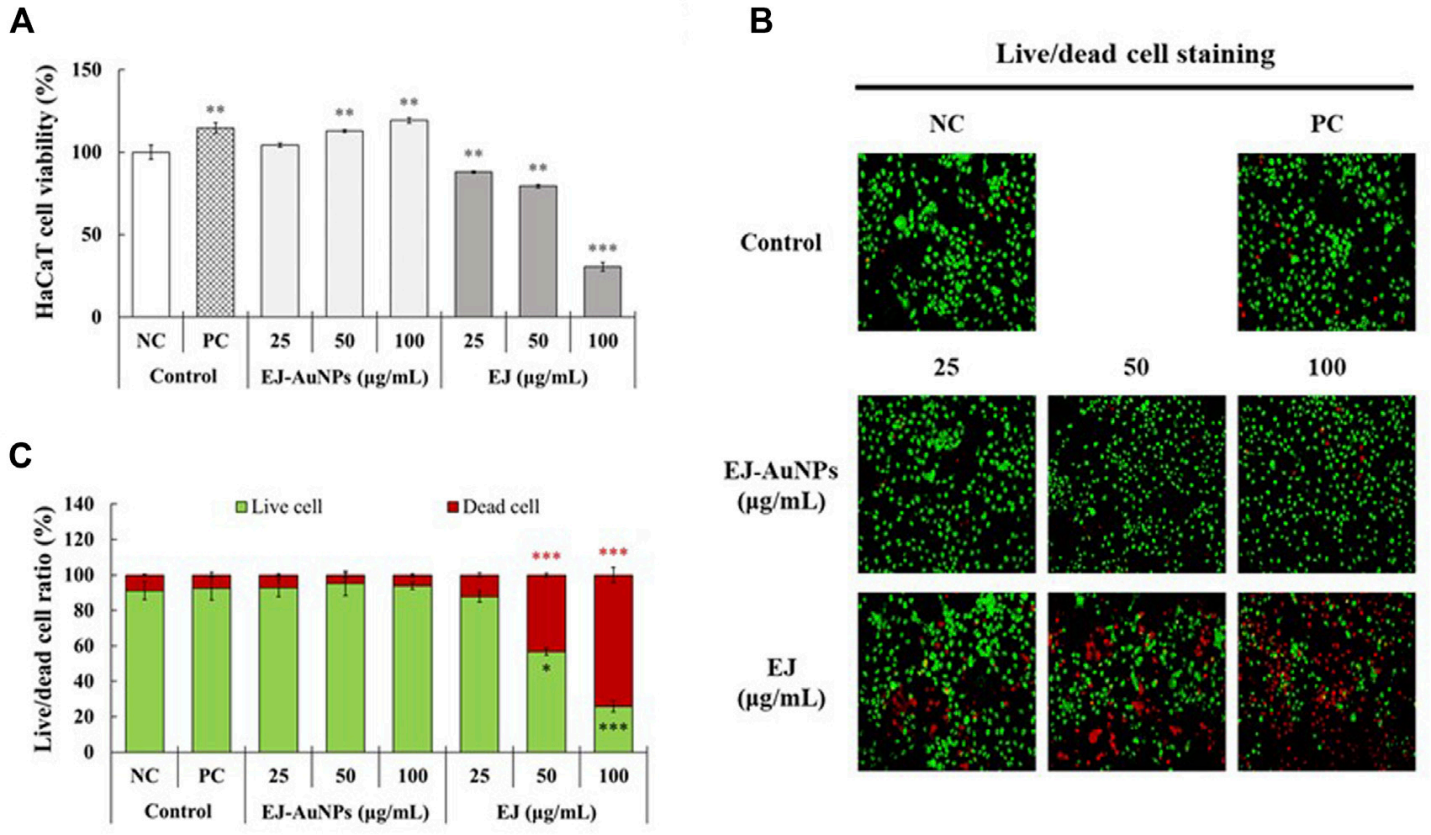
Cytotoxic effects of EJ and EJ-AuNPs against HaCaT keratinocytes. **(A)** The MTT assay. **(B)** Microscopic images and their **(C)** quantified results stained by live/dead cell staining dye. Live and dead cells were stained with green and red colors, respectively. NC, negative control treated with medium alone; PC, positive control treated with dexamethasone (20 μg/ml). Asterisks indicate the significant difference between NC and each group. *p < 0.05; **p < 0.01; ***p < 0.001.

The cytotoxic effects of EJ and EJ-AuNPs were evaluated and compared against normal HaCaT cells using a commercial MTT assay and live/dead cell staining. The MTT assay showed that EJ-AuNPs had no cytotoxic effect and even dose-dependently promoted cell proliferation (104.3–119.2%), compared to negative control (NC) cells (Figure 6A). However, significantly reduced viabilities were observed in EJ-treated cells by 12.0–69.4%, compared to NC cells. Figures 6B,C show the representative microscopic images of live/dead staining and their quantified results, respectively. The results showed that EJ-AuNPs did not induce a significant number of dead cells, but a significantly large number of cells were dead (12.1–74.1%) following EJ treatment, similar to the MTT result. These results suggest that EJ-AuNPs were considerably safer than EJ at equivalent concentrations in HaCaT cells.

### Effects of EJ-AuNPs on the expression levels of skin dermatitis-associated genes and protein secretion in T+I-induced HaCaT cells

**FIGURE 7:**
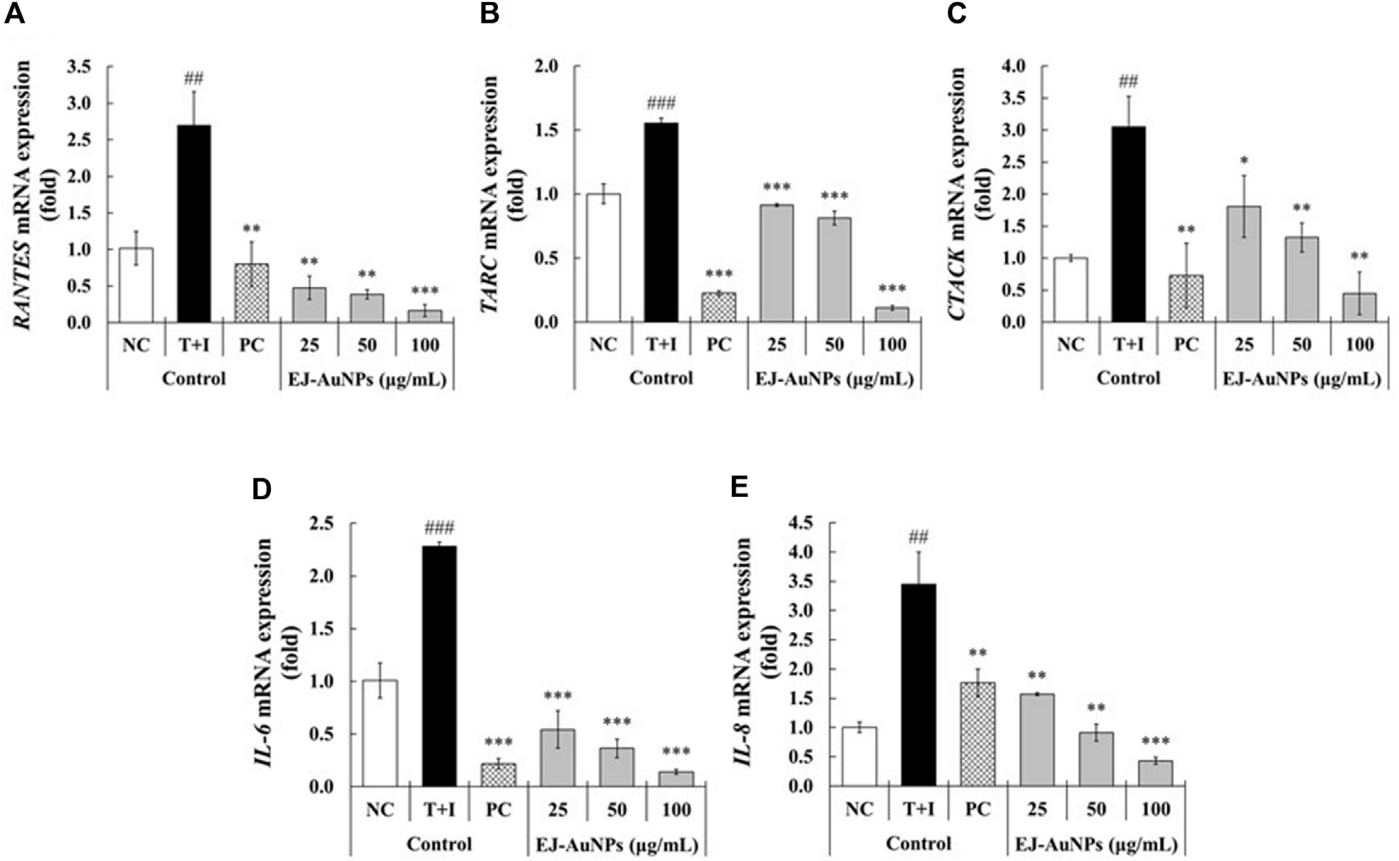
Effect of EJ-AuNPs on the expression levels of pro-inflammatory genes, including **(A)** C-C motif chemokine ligand 5 (*CCL5*)/regulated on activation, normal T cell expressed and secreted (*RANTES*), **(B)** thymus and activation-regulated chemokine (*TARC*/*CCL17*), **(C)** cutaneous T-cell attracting chemokine (*CTACK*/*CCL27*), **(D)** interleukin *(IL)-6*, and **(E)** *IL-8*/*CXCL-8*, in the tumor necrosis factor-alpha and interferon-gamma (TNF-α and IFN-γ; T+I)-treated HaCaT keratinocytes. NC, negative control treated with medium alone; T+I, inflammation-induced control treated with T+I alone; PC, positive control treated with dexamethasone (20 μg/ml) followed by T+I stimulation. EJ-AuNPs, sample groups treated with EJ-AuNPs followed by T+I stimulation. The crosshatch marks indicate the significant differences between NC and T+I, and asterisks indicate the significant differences between T+I and each group. *p < 0.05; ** and ^##^, *p* < 0.01; *** and ^###^, *p* < 0.001.

**FIGURE 8:**
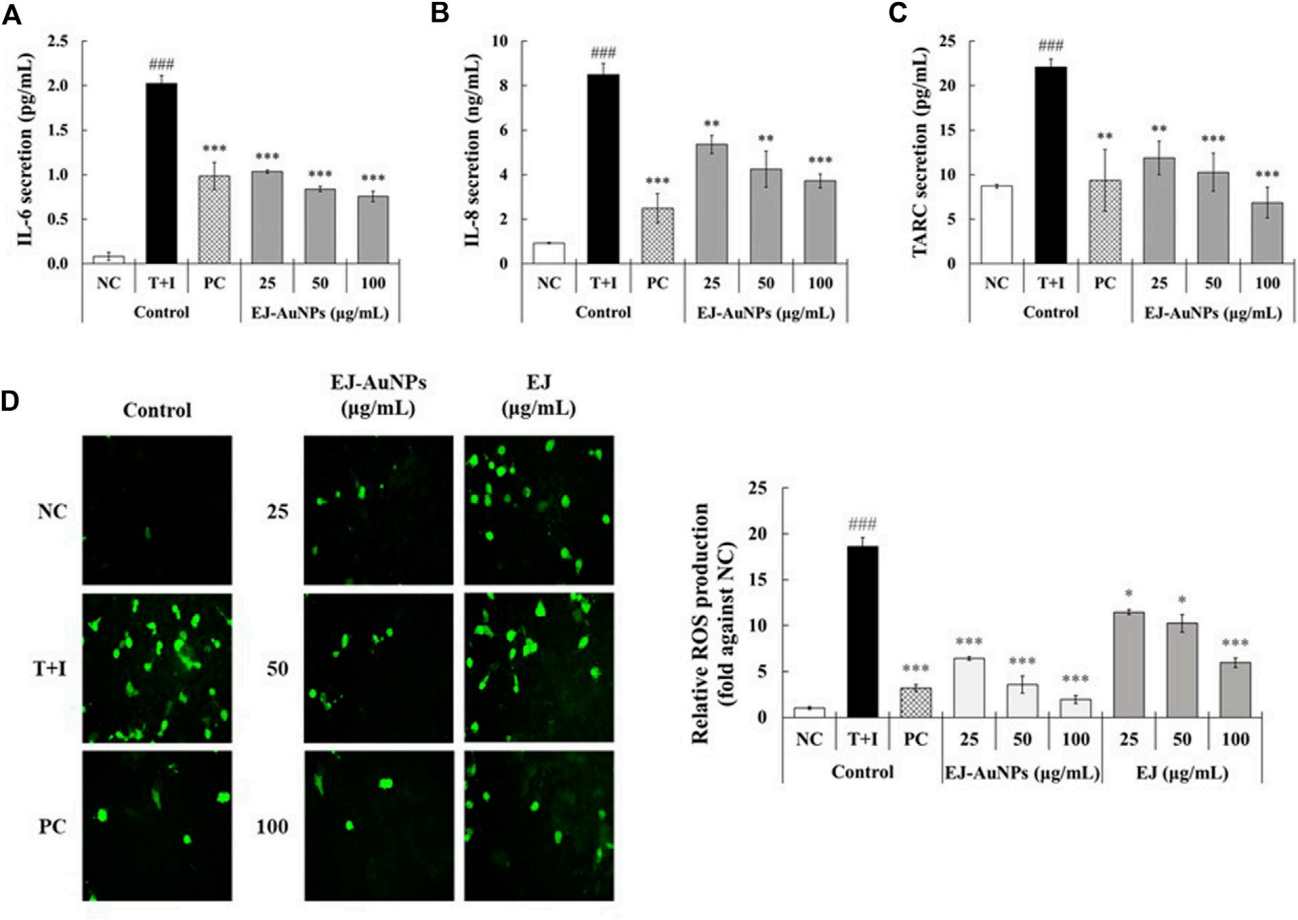
Effect of EJ-AuNPs on the secretion of pro-inflammatory cytokines, including **(A)** IL-6, **(B)** IL-8, and **(C)** TARC/CCL17, and production of intracellular **(D)** reactive oxygen species (ROS) in tumor necrosis factor-alpha and interferon-gamma (TNF-α and IFN-γ; T+I)-treated HaCaT keratinocytes. NC, negative control treated with medium alone; T+I, inflammation-induced control treated with T+I alone; PC, positive control treated with dexamethasone (20 μg/ml) followed by T+I stimulation. EJ-AuNPs, sample groups treated with EJ-AuNPs followed by T+I stimulation. The crosshatch marks indicate the significant differences between NC and T+I, and asterisks indicate the significant differences between T+I and each group. *p < 0.05; **p < 0.01; *** and ^###^, *p* < 0.001.

Figure 7 presents the results of skin dermatitis-associated mRNA expression determined by qRT-PCR. Compared to the NC group, excessive expression of pro-inflammatory chemokines, including the regulated on activation, normal T cell expressed and secreted (*RANTES*/*CCL5*), *TARC* (*TARC*/*CCL17*), cutaneous T-cell attracting chemokine (*CTACK*/*CCL27*), and *IL-8* (*CXCL8*)*,* and interleukin, *IL-*6, were induced in T+I-treated HaCaT cells. Dexamethasone treatment significantly decreased T+I-stimulated gene expression in HaCaT cells. Compared to the T+I group, the expression levels of *RANTES*, *TARC*, *CTACK*, *IL-6*, and *IL-8* genes were significantly downregulated by EJ-AuNP treatment. Next, we evaluated the inhibitory effects of EJ-AuNPs on skin dermatitis-associated cytokine secretion in T+I-induced keratinocytes using ELISA. As expected, T+I-stimulation induced significant secretion of IL-6, IL-8, and TARC in HaCaT keratinocytes, and dexamethasone treatment significantly decreased the level of T+I-stimulated secretion of these pro-inflammatory cytokines (Figures 8A–C). The increased levels of IL-6, IL-8, and TARC after T+I treatment were significantly decreased in a concentration-dependent manner following EJ-AuNP treatment. These results demonstrate that EJ-AuNPs effectively suppressed the skin dermatitis-associated mediators at the gene expression and protein secretion levels. We further tested the effect of EJ and EJ-AuNPs on the cellular ROS production capacity of T+I-treated HaCaT cells. As shown in Figure 8D, ROS production was significantly increased by T+I stimulation, and significantly decreased by dexamethasone treatment. Compared to T+I-treated cells, ROS production was significantly and concentration-dependently decreased by both EJ and EJ-AuNPs treatments, but higher reduction effects were observed in cells treated with EJ-AuNPs than in those treated with EJ.

### Identification of the molecular mechanism underlying skin dermatitis inhibition by EJ-AuNPs

**FIGURE 9:**
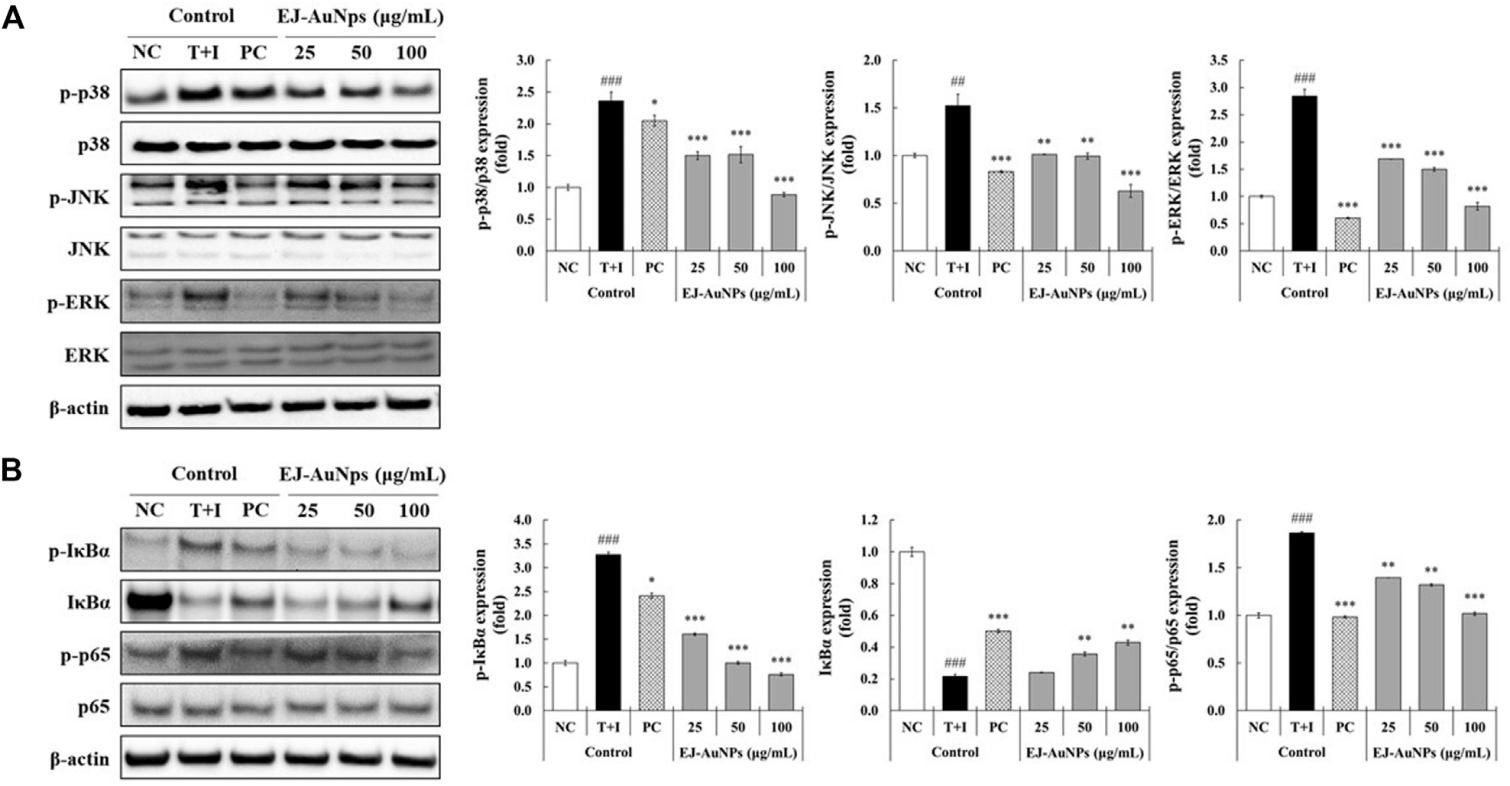
Effects of EJ-AuNPs on the **(A)** mitogen-activated protein kinase (MAPK) and **(B)** nuclear factor (NF)-κB signaling pathways in tumor necrosis factor-alpha and interferon-gamma (TNF-α and IFN-γ; T+I)-treated HaCaT keratinocytes. Results are presented as blotting images of expressed respective proteins and their quantification calculated by fold ratio of phosphorylated form/total form, except IκBα. NC, negative control treated with medium alone; T+I, inflammation-induced control treated with T+I alone; PC, positive control treated with dexamethasone (20 μg/ml) followed by T+I stimulation. EJ-AuNPs, sample groups treated with EJ-AuNPs followed by T+I stimulation. The crosshatch marks indicate the significant differences between NC and T+I, and asterisks indicate the significant differences between T+I and each group. *p < 0.05; ** and ^##^, *p* < 0.01; *** and ^###^, *p* < 0.001.

**FIGURE 10:**
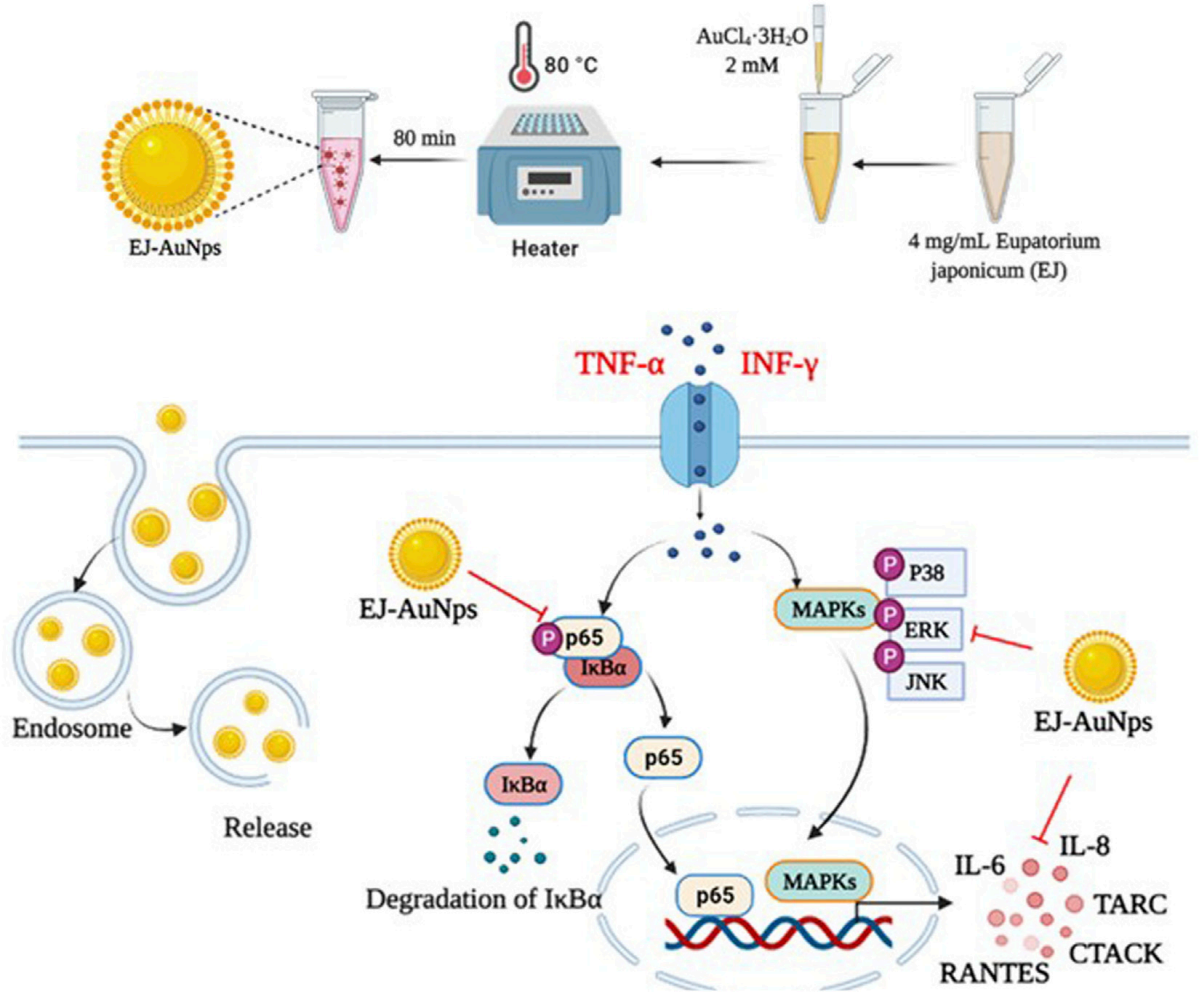
Proposed molecular mechanism of anti-inflammatory activity induced by EJ-AuNPs in TNF-α/IFN-γ-induced skin inflammatory HaCaT cells.

Next, we explored the signaling pathway associated with skin dermatitis inhibition by EJ-AuNPs in T+I-treated HaCaT cells. Figure 9 shows the western blotting images and quantification of phosphorylation associated with the mitogen-activated protein kinase (MAPK) (Figure 9A) and nuclear factor kappa-light-chain-enhancer of activated B cells (NF-κB) (Figure 9B) signaling pathways. T+I treatment significantly elevated the phosphorylation levels of three MAPKs (p38, JNK, and ERK) and NF-κB p65 without changing their corresponding total protein levels in HaCaT cells. Interestingly, T+I treatment significantly increased the phosphorylation levels of nuclear factor of kappa light polypeptide gene enhancer in B-cells inhibitor, alpha (IκBα), but significantly decreased the total protein levels. Following dexamethasone treatment, the levels of phosphorylated p38, JNK, ERK, IκBα, and p65 were significantly reduced, while the expression levels of intact IκBα were significantly upregulated. EJ-AuNP treatment significantly and concentration-dependently reduced the phosphorylation levels of three MAPKs (p38, ERK, and JNK) and two NF-κB-related molecules (IκBα and p65). In addition, the expression levels of intact IκBα were significantly upregulated by EJ-AuNP treatment in a concentration-dependent manner. Based on our finding, mechanism of action of anti-inflammatory activity mediated by EJ-AuNPs in TNF-α/IFN-γ-induced skin inflammatory HaCaT cells were proposed in Figure 10. These results demonstrate that the inhibitory effect on T+I-stimulated inflammation in HaCaT cells is closely associated with EJ-AuNP-mediated suppression of MAPK and NF-κB signaling.

## Discussion

According to recent studies, a number of natural plants have been shown to ameliorate ISDs (Yang et al., 2015; Lim et al., 2016; Yang et al., 2018); however, there are some concerns and limitations to the use of plant extracts and their active compounds in industrial applications due to their low solubility, stability, biocompatibility, and bioavailability (Kyriakoudi et al., 2021). Thus, studies aimed at overcoming these limitations are in high demand. It is well known that AuNPs that have unique physicochemical and multiple surface functionalities have several advantages, such as higher biodegradability, wide flexibility, improved stability, and lower possible adverse effects, facilitating their widespread use in biomedical field (Yeh et al., 2012). In the present study, we aimed to synthesize plant-loaded AuNPs by a biological process using the Korean endemic medicinal plant, EJ. First, the major secondary metabolites were confirmed as candidates for bioactive ingredients in the EJ extract using UHPLC equipped with electrospray ionization source (ESI) and MS/MS. As listed in Supplementary Table S1, data-dependent acquisition was accomplished using an ion trap mass spectrometer according to the previous method reported by Kim et al. (2021). Upon MS analysis, flavonoid glycosides, including melilotoside, rutin, hyperoside, nictoflorin, cynaroside, and rhamnetin, were putatively identified as the major phytochemicals in the EJ extract (Figure 1, Figure 2). Until now, only a few studies have been reported on either the physiological activity or active ingredients of EJ. With respect to the major chemical constituents, a previous study identified fatty alcohols, acids, terpenes, phytosterols, phenolic acids, and flavonoids in EJ leaves (Phan et al., 2021). However, the above-mentioned flavonoid glycosides confirmed in the present study appear to be the first identification of these components in EJ. Nevertheless, as MS analysis generally only facilitates the qualitative identification of putative compounds (Xiao et al., 2012), further studies are necessary for the quantitative identification of the above-mentioned compounds and other bioactive compounds in EJ.

Next, we established an optimal system for EJ-AuNP biosynthesis by monitoring the reaction conditions, such as EJ and gold salt concentrations, reaction pH, and time. Finally, EJ-AuNPs were biosynthesized under the optimized system (Figure 3). In general, the biosynthesis of AuNPs take place in two steps: reduction of Au^3+^ into Au^0^ and its agglomeration and stabilization to form the AuNPs (Sheny et al., 2011). Given that various secondary metabolites, such as phenolic acids, flavonoids, terpenoids, and polyphenols in the ethanolic extracts of plants can mainly participate in the AuNPs biosynthesis (Mikhailova, 2021), it can be speculated that aforementioned flavonoids in EJ may be responsible for the reduction in Au ions (Au^3+^ → Au^0^) and corresponding nanoparticles formation. Characterization of nanoparticles can be considered as a collection of analytical results obtained from the physical and chemical characteristics on the nanoparticles’ surface to create knowledge on synthesis, properties, and applications of the nanoparticles (Vijayakumar, 2019). Several microscopic and spectrometric technique were used in this study to measure, quantify, and image the surface properties of the synthesized EJ-AuNPs. Using those analyses, the nanoparticles were physicochemically characterized in terms of particle size, shape, and crystalline nature (Figure 4, Figure 5). A helpful information on the characterization techniques for nanoparticles can be available in recently published review article (Mourdikoudis et al., 2018). Consequently, the EJ-AuNPs were synthesized with various surface morphologies, such as circular, spherical, and polygonal shapes, with particle sizes of approximately 50–150 nm and no impurities. TGA result revealed that the synthesized EJ-AuNPs also showed better thermal stability than EJ, suggesting their potential for industrial applications. Furthermore, FT-IR analysis was performed to identify the presence of different functional groups in EJ and EJ-AuNPs at various positions. Although the biosynthetic mechanism of AuNPs would not been fully elucidated, several studies have reported FT-IR analysis is helpful to explore plant-derived AuNPs grafted on the surface characteristics and to presume the possible interactions of the AuNPs with different functional groups (Lokina et al., 2014; Ajitha et al., 2015; Khandanlou et al., 2020; Mikhailova, 2021). Our IR spectra displayed that EJ-AuNPs contain aromatic, hydroxyl, and carbonyl (aldehyde) groups as the functional groups which would have been derived from flavonoids, such as melilotoside, rutin, hyperoside, nictoflorin, cynaroside, and rhamnetin of EJ. A number of studies have reported that such flavonoids can be strongly involved in the reduction, capping, and stabilization processes in AuNPs biosynthesis (Ajitha et al., 2015; Khandanlou et al., 2020). It can be thus speculated that the functional groups, especially hydroxyl and carbonyl groups, in the aforementioned flavonoids present in EJ play an important role as reducing, capping, and stabilizing agents for EJ-AuNPs formation (Shankar et al., 2004; Sheny et al., 2011; Lokina et al., 2014).

The results of cytotoxic evaluation revealed that EJ-AuNPs induced the promoting effect of normal keratinocyte cell proliferation, although EJ showed a significant cytotoxic effect at equivalent concentrations, leading to the favorable safety of EJ-AuNPs when applied to human keratinocytes compared to EJ alone. This seems to be the first study to propose the possibility for lowering cytotoxicity of EJ by forming nanoformulation. From the subsequent experiments, except for EJ where cytotoxic effect was confirmed, only EJ-AuNPs were supposed to evaluate for anti-inflammatory activity.

We further investigated the effects of EJ-AuNPs on ISDs in an inflammatory epidermal keratinocyte model. Actually, during skin inflammatory reaction, epidermal keratinocytes that constitute nearly 90% of epidermal cells play a pivotal role in the progression and pathogenesis of dermatitis-related diseases by communicating with other cells (Lim et al., 2015; Kim et al., 2018). Keratinocytes and their secreted chemokines, primarily CC chemokine ligands (CCLs), including CCL5 (RANTES) (Yang et al., 2018), CCL17 (TARC) (Werfel, 2009), CCL22 (macrophage-derived chemokine, MDC) (Lim et al., 2015), CCL27 (CTACK) (Kakinuma et al., 2003), and CXC chemokine ligand (CXCLs, such as CXCL8 (IL-8) (Mirandola et al., 2011), are regarded as triggers for the progression of skin inflammatory diseases by mediating the recruitment of immune cells into the inflammatory skin tissue (Homey et al., 2006; Sugaya, 2015). In particular, these inflammatory chemokines can be highly expressed in keratinocytes when stimulated with macrophages or T cell-secreting cytokines, such as TNF-α and IFN-γ (Albanesi et al., 2005; Chieosilapatham et al., 2021). Accordingly, recent studies exploring the potential medications for ISDs have concentrated on the downregulation of these inflammatory chemokines and their underlying mechanisms of action in T+I-induced keratinocytes (Albanesi and Pastore, 2010; Lim et al., 2015; Yang et al., 2015; Juráňová et al., 2017). Our qRT-PCR and ELISA results demonstrated that EJ-AuNPs effectively suppressed the T+I-stimulated inflammatory chemokines (RANTES, TARC, CTACK, and IL-8) in addition to IL-6 in HaCaT keratinocytes (Figure 7, Figure 8). This suggests the possibility of using EJ-AuNPs as anti-inflammatory candidates against ISDs. In addition, the ROS staining results shown in Figure 8D indicate that these anti-inflammatory effects of EJ-AuNPs are highly associated with the suppression of T+I-stimulated intracellular ROS production in HaCaT cells. Excessive ROS accumulation can cause oxidative stress followed by critical damage of HaCaT cells (Shim et al., 2008; Han et al., 2021). Our results also suggest that EJ-AuNPs can be effective in protecting against this oxidative stress-mediated cell death in ISDs.

Cytokines are important mediators of the immune system, including the inflammatory response. Exposure of keratinocytes to Th cell-secreted cytokines, such as TNF-α and IFN-γ, triggers the undue production of inflammatory cytokines (mainly chemokines, e.g., CCL and CXCL), causing explosive immune responses via migration of leukocytes into inflammatory lesions in the skin (Homey et al., 2006; Nedoszytko et al., 2014). In particular, the MAPK and NF-κB cascades are the best-studied key signaling pathways within the cells involved in various inflammatory responses, such as the keratinocytes (Yang et al., 2015; Yang et al., 2018). MAPK signaling plays important roles in diverse pathological conditions and in the regulation of various cellular functions, including cell differentiation, proliferation, mitosis, survival, and death. Recent studies have reported that mitogenic and proliferative stimuli favorably activate the ERK pathway, whereas inflammatory and environmental stresses preferentially stimulate the activation of p38 and JNK (Khorasanizadeh et al., 2017). Nevertheless, three distinct MAPKs are considered to be key target molecules for the treatment of various diseases, including allergic inflammatory diseases (Khorasanizadeh et al., 2017; Sur et al., 2019). Recent studies have proposed that the nanoparticle-mediated targeting of MAPK is an attractive therapeutic strategy for treating various diseases (Basu et al., 2009; De Araújo Jr et al., 2018). NF-κB is closely involved in the pathogenesis of various inflammatory diseases, such as asthma, bronchitis, colitis, lupus vulgaris, and sclerosis (atherogenic and multiple). NF-κB family of inducible transcription factors are composed of five subfamilies: NF-κB1 (p105/p50), NF-κB2 (p100/p52), RelA (p65), RelB, and c-Rel (Caamaño and Hunter, 2002; Liu et al., 2017). In unstimulated stages, NF-κB proteins are sequestered by a family of their inhibitor proteins, such as IκBα, IκBβ, and IκBγ (Liu et al., 2017). When cells are stimulated by various external stimuli, the inactive form of the NF-κB/IκB complex is activated by IκB phosphorylation and released from the NF-κB protein (Liu et al., 2017). The liberated NF-κB proteins translocate into the nucleus to promote the inflammatory response by inducing the transcription of proinflammatory genes (Shin et al., 2021). To explore the intracellular mechanism underlying EJ-AuNP-driven anti-inflammatory effects, the MAPK and NF-κB signaling pathways were investigated. Figure 9 indicates that the phosphorylation of three MAPKs (ERK, JNK, and p38) and two NF-κB signaling molecules (IκBα and p65) was markedly inhibited by EJ-AuNPs treatment. Taken together, our study demonstrates that the inhibitory effects of EJ-AuNPs on T+I-induced chemokine and cytokine production are significantly associated with the downregulation of the MAPK and NF-κB intracellular signaling pathways. However, the present study has a limitation to be addressed in the further work. That is, the safety and anti-inflammatory efficacy of EJ-AuNPs should be evaluated by *in vivo* system to clearly elucidate their effectiveness and increase their possibility of using as anti-inflammatory agents industrially.

## Conclusion

Until now, studies have rarely reported on the application of plant-derived AuNPs for the skin health. To investigate the possibility of using the plant-derived AuNPs as anti-dermatitis candidate, the present study was aimed to prepare novel AuNPs using EJ extracts and evaluate their physicochemical characteristics, anti-inflammatory efficacy, and molecular mechanism. Novel EJ-based gold nanoparticles (EJ-AuNPs) were successfully prepared using a green synthesis method, and the optimized conditions and physicochemical properties of the EJ-AuNPs were characterized using various microscopic and spectrometric analyses. In an *in vitro* skin inflammation model using T+I-induced HaCaT cells, the synthesized EJ-AuNPs inhibited the activation of MAPK and NF-κB signaling, and the inhibition of T+I-stimulated inflammatory mediators (ROS, chemokines, and cytokines) may be associated with the suppression of these pathways. Our study offers preliminary results and a valuable strategy for the development of novel anti-skin dermatitis candidates using plant extract-based AuNPs. The study also suggests a possibility that biological green synthesis of gold nanoparticles from EJ may be useful for exerting anti-inflammatory potential while reducing cytotoxic effect of EJ. However, the present study has a limitation to be addressed in the further work. That is, the safety and anti-inflammatory efficacy of EJ-AuNPs should be evaluated by *in vivo* system to clearly elucidate their effectiveness and increase their possibility of using as anti-inflammatory agents industrially.

## Data Availability

The original contributions presented in the study are included in the article/Supplementary Material, further inquiries can be directed to the corresponding authors.
