# Supplementary material for: Structural properties and anti-dermatitis effects of flavonoids-loaded gold nanoparticles prepared by Eupatorium japonicum
Source: Front Pharmacol. 2022 Oct 31;13:1055378. doi: 10.3389/fphar.2022.1055378 (PMC9659597; doi:10.3389/fphar.2022.1055378)
Supplement: Supplementary file 1 [file Table1.DOCX]

Supplementary Material

## Supplementary Tables

# Table S1. Analytical conditions for UHPLC-MS/MS

| UHPLC system | LTQ Orbitrap XL (Thermo Electron Co., Waltham, MA, USA) |
| --- | --- |
| Stationary phase | ACQUITY UPLC BEH C18 column (2.1×150 mm, 1.7 μm; Waters, Co., Milford, MA, USA) |
| Mobile phase and gradient program | 1. 0.1% formic acid/water 2. 0.1% formic acid/acetonitrile  - -3 min → 0 min (5% B, equilibrium) - 0 min → 0.5 min (5% B) - 0.5 min → 10 min (5% B → 80% B) - 10 min → 10.1 min (80% B → 100% B) - 10.1 min → 12 min (100% B) |
| Ionization | Heated electrospray ionization (HESI) at 300°C |
| Spray voltage | 5.0 kV |
| Nebulizer sheath and auxiliary gases | 50 and 5 arbitrary unit (arb) |
| Mass range | 100–1000 *m/z* |
| Collision-induced dissociation (CID) | 45% |
| Data acquisition | Xcalibur (Thermo Electron Co., Waltham, MA, USA) |

**Table S2. Gene-specific primer sequences used in this study**

| IL-6 | Forward:  Reverse: | 5'–GTTCTCTGGGAAATCGTGGA–3'  5'–TGTACTCCAGGTAGCTATGG–3' |
| --- | --- | --- |
| IL-8 | Forward:  Reverse: | 5'–GAGAGTGATTGAGAGTGGACCAC–3'  5'–CACAACCCTCTGCACCCAGTTT–3' |
| RANTES/CCL5 | Forward:  Reverse: | 5'–CATATTCCTCGGACACCACACCCT–3'  5'–ACTCCTGACCTCAAGTGATCCACC–3' |
| TARC/CCL17 | Forward:  Reverse: | 5' –TGTAAAACGACGGCCAGT–3'  5'–CAGGAAACAGCTATGACC–3' |
| CTACK/CCL27 | Forward:  Reverse: | 5'–CACTGCCTGCTGTACTCAGCTCTA–3'  5'–CTTCAGCCCATTTTCCTTAGCATC–3' |

## Supplementary Figures

**A
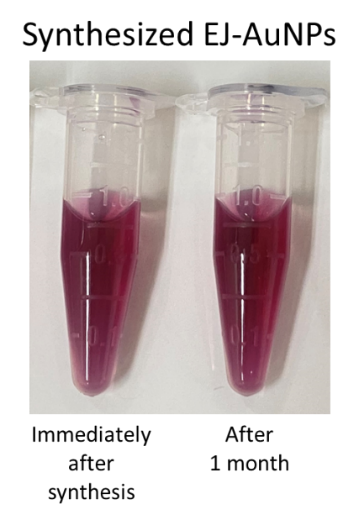
 B
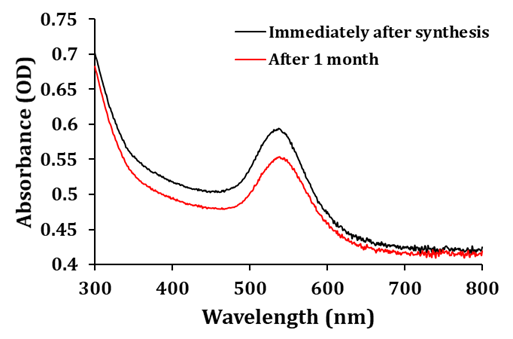
**

**C
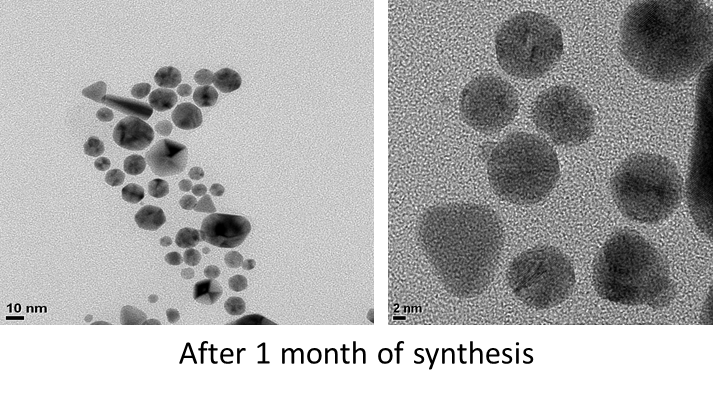
**

**Figure S1.** (A) No color changes or decomposed materials were observed in the synthesized EJ-AuNPs, when they stored at 25 °C for 1 month. (B) Scanning spectra at absorbances of 300–800 nm of the synthesized EJ-AuNPs. (C) Transmission electron microscopy (TEM) images of the synthesized EJ-AuNPs.
